# Pubertal Physical Activity and Cardiorespiratory Fitness in Relation to Late Adolescent Body Fatness in Boys: A 6-Year Follow-Up Study

**DOI:** 10.3390/ijerph18094881

**Published:** 2021-05-03

**Authors:** Liina Remmel, Reeli Tamme, Vallo Tillmann, Evelin Mäestu, Priit Purge, Eva Mengel, Eva-Maria Riso, Jaak Jürimäe

**Affiliations:** 1 Institute of Sport Sciences and Physiotherapy, Faculty of Medicine, University of Tartu, 50090 Tartu, Estonia; liina.remmel@ut.ee (L.R.); evelin.maestu@ut.ee (E.M.); priit.purge@ut.ee (P.P.); eva-maria.riso@ut.ee (E.-M.R.); 2 Institute of Clinical Medicine, Faculty of Medicine, University of Tartu, 50406 Tartu, Estonia; reeli.tamme@kliinikum.ee (R.T.); vallo.tillmann@kliinikum.ee (V.T.); evamengel@nooruse.ee (E.M.); 3 Department of Physiotherapy and Healthcare, Tartu Health Care College, 50411 Tartu, Estonia

**Keywords:** physical activity, cardiorespiratory fitness, body fatness indices, tracking, puberty, adolescence

## Abstract

There is a lack of studies investigating whether objectively measured physical activity (PA) and cardiorespiratory fitness (CRF) in puberty is associated with healthier body composition in late adolescence. The study sample included 71 boys, who were measured at puberty (mean age of 12.1 years) and again at late adolescence (mean age of 18.1 years). Physical activity was measured by accelerometry, and total PA, moderate-to-vigorous PA (MVPA), and sedentary time (SED) were calculated, while CRF was assessed by a peak oxygen consumption test. Body composition was measured by dual-energy X-ray absorptiometry, and body fat percentage (%BF), fat mass index (FMI), trunk fat mass index (TFMI), and fat-free mass index (FFMI) were calculated. Body mass index (BMI) and SED time increased, while MVPA, total PA, and CRF decreased from puberty to late adolescence. A relatively high degree of tracking for BMI and CRF, and a low tracking for PA from puberty to late adolescence was observed. Only the CRF value at puberty negatively predicted adolescent BF% (β = −0.221; *p* = 0.015), FMI (β = −0.212; *p* = 0.006), and TFMI (β = −0.189; *p* = 0.015) values. Adolescents whose CRF was above the median at puberty had lower BMI, BF%, FMI, and TFMI in comparison to those whose CRF was below the median at puberty. In conclusion, measured CRF and PA values decreased from puberty to late adolescence. Pubertal CRF predicted body fatness indices six years later in healthy adolescents.

## 1. Introduction

Childhood obesity is a serious public health concern globally, with obese children typically being less fit and physically active [1,2]. Childhood obesity has been found to track strongly into adulthood together with other cardiovascular disease risk factors [3]. It has to be considered that a sedentary lifestyle and poor physical fitness carry various unfavourable health effects [4]. Therefore, higher levels of physical fitness have been found to counteract some of these negative health effects of obesity [1], and has been suggested to be a powerful marker of health in children and adolescents [5]. It appears that physical fitness tracks rather well from childhood into adolescence, while physical activity (PA) demonstrates weaker stability during this period of life [6,7]. There are also studies demonstrating no tracking of leisure-time PA from the age of 9 to 17 years [4], and the relationship between PA and physical fitness can be rather weak during growth in childhood [4,8].

The interrelationships among PA, physical fitness, and various health outcomes are well-established in adults [9,10]. Higher levels of PA and cardiorespiratory fitness (CRF) are associated with lower body fatness levels in children [11,12] and adolescents [5,13], and have been suggested to decrease the risks associated with obesity and other cardiovascular disease risk factors later in life [9,14]. In addition, it has been demonstrated that higher PA and physical fitness levels are longitudinally associated with lower body fatness levels in children [15,16,17] and adolescents [13,18]. For example, moderate-to-vigorous PA (MVPA) levels in 12-year-old children were strongly and inversely associated with body fat mass (FM) two years later [19], while higher CRF levels in 4.5-year-old children were related to lower body FM and higher fat-free mass (FFM) one year later [20]. In addition, changes in CRF were negatively associated with changes in body fat percentage (BF%) and FM index (FMI) in children in the transition from preschool to school [16]. In contrast, baseline MVPA levels were not associated with waist circumference in children and adolescents at two years’ follow-up [21]. The evidence showing longitudinal associations among childhood PA and/or fitness with adult body composition is relatively scarce [15,22,23]. For example, adolescent CRF level was related to adult body fatness indicators in healthy subjects over an 11-year study period [22]. However, most of the above-described studies have used indirect measures for some predictive and outcome parameters, whereas the application of objective parameters can yield more reliable results [18]. Accordingly, the current study used an objective assessment of PA by accelerometry, while CRF was directly assessed by peak oxygen consumption (VO_2_peak) and body composition indices by dual-energy X-ray absorptiometry (DXA). The main purpose of this study was to examine the associations of PA and CRF in puberty with body composition indices six years later in late adolescence. 

## 2. Materials and Methods

### 2.1. Research Design and Participants

This study conducted in 2017–2018 was a follow-up of a longitudinal research project carried out between 2009–2013, and complete details and participants of the project are available in our previous studies [13,18,24,25,26,27,28]. The participants of this longitudinal study were Estonian boys, recruited from Tartu City and County. Only boys who reported that they were healthy without any additional illnesses (any current acute or chronic illnesses) and were allowed to attend obligatory physical education classes in school (no health-related problems, injuries, etc.) were recruited in the study. At baseline, 313 boys took part in this investigation. Baseline data collection took place in 2010–2011. The invitation to participate in the current investigation was sent to all 217 individuals who had participated in 12-month and 24-month follow-ups in 2011–2013. Of these, 88 individuals participated in the follow-up at the mean age of 18.1 years. Bone health, PA, and blood hormones over the six-year period have already been published [29,30]. Valid and most complete data about CRF, PA, and body composition were available for 71 subjects. Accordingly, the final number of participants included in the analysis was 71. All 71 boys were at pubertal stages 2 and 3 at the first measurement occasion, and at pubertal stage 5 at the follow-up measurement, as determined by a self-reported questionnaire of pubertal stages according to Tanner [31]. There were no differences in the age, anthropometric, and body composition data between subjects at the late adolescence measurement who were included in the final analysis (*n* = 71) and those who were not (*n* = 17). 

The study was conducted following the Declaration of Helsinki, and the protocol was approved by the Research Ethics Committee of the University of Tartu, Estonia (Consent No 260/t-19). All participants and their parents were thoroughly informed about the purpose and consent of the study, and signed written informed consent was obtained from all participants before entering the study. When the participant was younger than 18.0 years, additional signed informed consent was retrieved from his parent before participation [29,30]. 

Measurements of the current investigation included anthropometry, PA, and CRF at both measurement times. In addition, body composition was measured by DXA and body composition indices computed at the follow-up measurement in late adolescence.

### 2.2. Measurements

#### 2.2.1. Anthropometry and Body Composition

Body height was measured to the nearest 0.1 cm using Martin’s metal anthropometer according to the standard technique (GMP Anthropological Instruments, Zurich, Switzerland). Body mass was measured to the nearest 0.05 kg using medical scales (A&D Instruments Ltd., Abington, UK). Body mass index (BMI; kg/m^2^) was calculated as body mass (kg) divided by height squared (m^2^). In addition, DXA (Hologic QDR Series, Waltham, MA, USA) was used to measure total body fat mass (FM), total body fat-free mass (FFM), and trunk FM at the follow-up measurement occasion. The coefficients of variation for these body composition variables were less than 2%. The standard subject positioning was used for total body measurements, and participants were scanned in light clothing while lying flat on the back, with arms at the sides [25]. As the significance of the BMI is not clear as body mass is composed of two distinct components (i.e., FFM and FM), FFM index (FFMI; kg/m^2^) and FM index (FMI; kg/m^2^) were also calculated [16,17,24]. In addition, trunk fat mass index (TFMI; kg/m^2^) was calculated as trunk FM divided by height squared, and was used as an indicator of body fat distribution [32]. 

#### 2.2.2. Cardiorespiratory Fitness

Cardiorespiratory fitness (CRF) was determined by a stepwise incremental exercise test until volitional exhaustion using an electrically braked bicycle ergometer (Corival V3; Lode, The Netherlands). At baseline, the initial work rate was 40 W, which was increased to 50 W during the second measurement time. The stage increments were 35 W and 50 W after every 3 min during baseline and the second measurement time, respectively [18]. Although changes in assessment protocol were made between years, the testing could still be compared in terms of fitness levels [18,33,34]. The test was designed to elicit maximal power output at approximately 15–18 min for each subject [18,33,34]. Pedaling frequency was set to 60–70 rpm. Respiratory gas exchange variables were measured throughout the test using breath-by-breath mode with data being recorded in 10 s intervals. The averaging of 10 s sampling intervals was used by standard software. During all tests, subjects breathed through a facemask. Oxygen consumption, carbon dioxide output, and minute ventilation were continuously measured using a portable open-air spirometry system (MetaMax I, Cortex, Leipzig, Germany). The analyzer was calibrated with gases of known concentration before the test, according to the manufacturer’s guidelines. All data were calculated by means of computer analysis using standard software (MetaMax-Analysis 3.21, Cortex, Leipzig, Germany). Peak oxygen consumption (VO_2_peak; L/min) was measured, and CRF was defined as the VO_2_peak per kilogram of body mass (mL/min/kg) [18,33,34]. Participants were verbally encouraged to produce maximal effort. The highest 10 s VO_2_ attained was accepted as a peak index if clear signs of intense exertion (e.g., hyperpnoea, facial flushing, unsteady gait, and profuse sweating) were demonstrated and supported by a respiratory exchange ratio greater than 1.00, and a heart rate which was levelling-off over the final stages of the test at a value within 5% of the mean peak heart rate that has previously been reported for similar test protocol [18,35]. 

#### 2.2.3. Physical Activity

Physical activity (PA) was objectively assessed by accelerometer model GT1M at baseline in puberty and model GT3X at a six-year follow-up in late adolescence (ActiGraph LLC, Pensacola, FL, USA) designed to register vertical accelerations [29]. Each participant was asked to wear the accelerometer on their right hip for seven consecutive days during the wake-up time, except in conditions where the accelerometer could get wet (e.g., swimming, sauna, bathing). The accelerometer was programmed to record activity counts in 15-s epochs [13,18,36]. For the analyses of accelerometer data, all night activity (24:00–6:00 h) and all sequences of consecutive zero counts of 10 min or more were excluded from each individual’s record. At least two weekdays and one weekend day of recording with a minimum of 10 h/day was set as an inclusion criterion [13,18,36]. Data were downloaded from the accelerometer and processed using ActiLife software version 6.11.2 (ActiGraph LLC, Pensacola, FL, USA). Summary variables of daily min of sedentary time (SED; min/day), moderate-to-vigorous PA (MVPA; min/day), and total PA (counts/min) were determined as described previously [13,18,36]. Specifically, the following cut-offs were used: SED ≤ 100 counts/min, moderate PA 2296-4011 counts/min, and vigorous PA ≥ 4012 counts/min [37]. The time spent in at least moderate PA intensity was calculated as MVPA, while the total PA was expressed as the total number of counts divided by the registered time (counts/min) [13,18,36].

### 2.3. Statistical Analysis

Data analysis was performed using the SPSS software (version 21.0 for Windows; SPSS Inc., Chicago, IL, USA). Standard statistical methods were used to calculate means and standard deviations (±SD). All variables were checked for normality of distribution before the analysis. A paired samples *t*-test was used to determine differences in mean values between baseline and follow-up [29,30]. Pearson correlation coefficients were used to study the tracking of measured variables at baseline and follow-up measurements [36], while Spearman correlation analysis was conducted to describe the relationships between pubertal PA and CRF variables with late adolescent body composition indices [29,30]. In addition, linear regression analysis was applied to examine associations of PA and CRF variables at baseline with body composition indices in follow-up after adjusting for potential confounders such as baseline age, BMI, total PA, and total accelerometer awake wear time [16]. The results of the regression models are reported as the standardized regression coefficient (β). The variance inflation factors between variables were less than five, denoting that collinearity was not a concern [16]. Finally, pubertal CRF, MVPA, and BMI indices were split into two categories based on the median split: low (below median) and high (above median) to further examine these associations [22]. Significance was set at *p* < 0.05.

## 3. Results

The characteristics of 71 boys at baseline (average age: 12.1 ± 0.6 years) and late adolescence (average age: 18.1 ± 0.6 years) are shown in Table 1. The average follow-up time from puberty to late adolescence was 5.9 ± 0.3 years. It appeared that six boys at baseline (8%) and eight boys at late adolescence (11%) were overweight. As expected, height, body mass, BMI, and SED values significantly increased (*p* < 0.05), while MVPA, total PA, and CRF significantly decreased (*p* < 0.05) from puberty to late adolescence (Table 1). All tracking coefficients between pubertal and late-adolescent BMI, SED, MVPA, total PA, and CRF values over the six-year period were significant (*p* < 0.05). The tracking of BMI was high (r = 0.826). Tracking coefficients for PA indices (MVPA: r = 0.297; total PA: r = 0.356) and SED time (r = 0.250) were low, while the tracking for CRF was moderate (r = 0.532). These tracking coefficients demonstrate a relatively high degree of tracking for BMI and CRF variables and relatively lower tracking of PA and SED indices from puberty to late adolescence.

Associations of PA and CRF indicators at puberty with body composition indices at late adolescence are presented in Table 2 and Table 3. Pubertal CRF, but not total PA, MVPA, or SED time was negatively correlated (*p* < 0.05) with late-adolescent body fatness indicators (BMI, BF%, FMI, and TFMI) (Table 2). After adjustments for age, BMI, total PA, and total awake accelerometer wear time at puberty, the negative association between CRF in puberty and BF%, FMI, and TFMI values at late adolescence still remained significant (*p* < 0.05) (Table 3).

When subjects were divided by their CRF values at puberty into two groups, above and below median, the former group had significantly lower BMI, FMI, and TFMI indices compared to those in the below-median group (Table 4). In addition, adolescents whose BMI values were above the median at puberty had higher body composition indices (BF%, FMI, TFMI, and FFMI) compared with adolescents whose BMI values were below median at puberty. No such differences were seen in assessed SED time and PA indices. When subjects were divided by their MVPA values at puberty into two groups, above and below median, no such differences were seen in BMI, BF%, FMI, TFMI, or FFMI indices.

## 4. Discussion

The main aim of the current study was to examine the associations of measured PA and CRF values in 12-year-old boys with body composition values recorded six years later, when the boys reached a mean age of 18 years. Another aim was to study the tracking of BMI, PA, and CRF variables over a six-year period in healthy adolescent boys. The results of the current study demonstrated that baseline CRF was an important pubertal parameter to characterize assessed body fatness indices six years later in healthy adolescent boys. In addition, the tracking coefficients of BMI and CRF showed a higher degree of tracking, while relatively low tracking coefficients were found for PA and SED indices over the study period.

Cardiorespiratory fitness has been suggested to be a powerful marker of health in children and adolescents [5]. The moderate tracking coefficient of CRF (r = 0.532) over the six-year period during adolescence in studied healthy boys was similar to previous findings in healthy adolescents during the transition from childhood to adulthood [22]. This stability in measured CRF over a relatively long period during growth together with the finding that those pubertal boys with high CRF values showed lower levels of body fatness indices as late adolescents suggests that pubertal fitness is an important marker of healthy body composition later in life. Similarly to our results, higher physical fitness levels have been found to be longitudinally associated with lower body fatness levels during childhood [13,15,16], while Eisenmann et al. [22] demonstrated that adolescent CRF level predicted adult body fatness indicators in healthy subjects over an 11-year study period. In their study, CRF was assessed by a maximal treadmill running time test and not directly measured by a VO_2_peak test [22]. Another study by Lima et al. [15] also demonstrated that CRF measured directly using VO_2_peak test, and not motor competence nor PA variables, had the strongest longitudinal impact on body fatness across a seven-year period in initially six-year-old children. In accordance with our results, Rodrigues et al. [38] investigated schoolchildren from 9 years till the age of 15 years and found that a negative developmental pathway in CRF increased, several times, a chance to become overweight or obese at the age of 15 years compared to children with a positive developmental pathway of CRF development [38]. Taken together, the results of our study extend this assumption and demonstrate that CRF measured directly by a VO_2_peak test and not objectively measured PA variables in initially 12-year-old boys was also the strongest predictor of body fatness indices at 18 years of age.

Decreases in measured PA variables and increases in SED time over the six-year study period are in accordance with other longitudinal studies in similar age groups [39,40,41,42]. In accordance with our results, a recent study by Lagestad et al. [40] using accelerometers showed a substantial decrease in MVPA levels from 67 until 24 min/day during the five-year period from age 14 to 19, while in our study, the decrease in MVPA levels was from 69 to 58 min/day during the six-year period from age 12 to 18. The tracking of PA variables was relatively low over the six-year period in our study, while moderate tracking coefficients for measured PA variables have been reported in different longitudinal studies using shorter time-periods in adolescents [36,39]. Interestingly, the tracking of SED time was relatively low in our study, while there are studies to demonstrate better tracking for inactivity than for activity in younger ages [6]. However, according to the results of our study, the increase in SED time from puberty to late adolescence is still noticeable and it has been suggested that prevention strategies at this period of life should concentrate on the reduction of SED time [42]. The increase in SED time during this period of life is partly due to the more demanding academic/work requirements compared to childhood and also to physiological changes leading to less spontaneous PA in comparison with childhood [41]. However, SED time was not associated with body fatness measures from childhood through to young adulthood [23]. In contrast, growth models indicated that low levels of MVPA, but not high levels of SED time were associated with high levels of body fatness in male subjects, and it was argued that public health approaches should focus on increments in MVPA rather than decrements in SED time [23]. 

Previous longitudinal studies have demonstrated that accelerometry-assessed PA values predicted different body fatness values in children [13,19,42], while there are also investigations to show that PA did not predict later adiposity in children and adolescents [21,43]. In our study, none of the measured PA variables at puberty was associated with later body composition indices in studied males. Furthermore, no differences in late adolescent body composition and CRF values were seen between subjects belonging to high or low pubertal MVPA groups (see Table 4). Our longitudinal results clearly demonstrate that pubertal MVPA does not directly contribute to body composition indices in late adolescence. In accordance with our results, Hjorth et al. [43] found that none of the measured PA variables at baseline predicted changes in FMI, while higher FMI at baseline predicted a decrease in total PA and MVPA, and an increase in SED time. Accordingly, it could be suggested that pubertal PA does not have a direct impact on body fatness indices later in adolescence. Instead of this, pubertal CRF had a direct influence on body fatness measures in late adolescence in our studied boys. In accordance, Lima et al. [15] also argued that CRF exhibited independent longitudinal association with body fatness, while PA only influenced body fatness through CRF in children over a seven-year measurement period. 

The results of our study demonstrated that BMI increased during the study period, which is in agreement with other longitudinal studies in a similar age range [22,40]. However, this increase in BMI in our study does not manifest negative health outcomes with regard to this increase in relation to body fatness, although the negative effect of BMI increase in this age range has been highlighted [40]. Therefore, the tracking of BMI was high (r = 0.826) over the six-year study period from puberty to late adolescence, indicating remarkable stability over this period of life. It has been demonstrated previously that body fatness indicators are moderately stable (tracking coefficients ranging from 0.44 to 0.78) over 11 years in males of normal weight from puberty to young adulthood [22]. Our results also showed that directly measured VO_2_peak as a marker of CRF decreased between the ages 12 to 18 years, similarly to the study of Lagestad et al. [40] in the same age range, which is a negative finding in terms of health. Poor CRF is a strong longitudinal predictor of cardiovascular disease risk factors in adolescent boys [18].

A major strength of this study is that it was based on a longitudinal design, where the same participants were monitored from 12 to 18 years of age [29,30]. In addition, the main variables measured, such as PA, VO_2_peak, and body composition, were based on high-quality standard procedures. It is also important that FMI determined by DXA was used over BMI to characterize body fatness indices in late adolescence in studied males. The major limitation of the study is the fact that body composition was measured by DXA only in the follow-up measurement at late adolescence. This did not allow to determine CRF as the VO_2_peak per kilogram of FFM, which is the more relevant morphological variable than body mass in the development of CRF during growth and maturation [35]. However, similarly to our results, CRF has been determined as the VO_2_peak per kilogram of body mass in different longitudinal studies with children and adolescents [4,6,18,33,34]. Another limitation is the relatively small sample size due to the low participation rate of subjects. The relatively long study period and the participants’ movement to other cities after finishing secondary school would be the most likely reason for that. The difficulties in conducting longitudinal studies from childhood into adulthood are well-recognized [22]. However, the sample size in our study was comparable with other studies in this area [22,40]. In addition, our results are limited to boys. A similar study with females is needed to clarify whether longitudinal associations of cardiorespiratory fitness with body fatness indices is also relevant for girls progressing from puberty to late adolescence. Finally, this is an observational study which does not prove causality. 

## 5. Conclusions

Higher level of pubertal cardiorespiratory fitness, and not physical activity, was associated with lower body fatness indices in late adolescence in a sample of healthy males. Body mass index and cardiorespiratory fitness values showed a relatively high degree of tracking, while the tracking of physical activity measures was relatively low over the six-year period from puberty to late adolescence. Finally, measured cardiorespiratory fitness and physical activity values decreased during this period of life.

## Figures and Tables

**Table 1:** Mean (±SD) participant anthropometric, physical activity intensities, and cardiorespiratory fitness characteristics over the six-year study period.

Variable	Puberty	Late Adolescence	*p* Value
Age (years)	12.1 (0.6)	18.1 (0.6)	<0.0001
Height (m)	1.55 (0.08)	1.81 (0.07)	<0.0001
Body mass (kg)	47.3 (12.5)	73.4 (11.0)	<0.0001
BMI (kg/m^2^)	19.5 (3.9)	22.4 (3.0)	<0.0001
SED (min/day)	531.1 (63.9)	633.7 (86.1)	<0.0001
MVPA (min/day)	69.0 (26.7)	57.9 (26.5)	0.004
Total PA (counts/min)	475 (153)	394 (129)	<0.0001
CRF (mL/min/kg)	51.0 (8.2)	45.3 (8.2)	<0.0001

BMI, body mass index; SED, sedentary time; PA, physical activity; MVPA, moderate-to-vigorous PA; CRF, cardiorespiratory fitness.

**Table 2:** Simple correlations of physical activity and cardiorespiratory fitness variables in puberty (mean age of 12.1 years) with body composition indices determined by dual-energy X-ray absorptiometry in late adolescence (mean age of 18.1 years).

Pubertal Variable	Late Adolescent Variable				
	BMI (kg/m^2^)	BF%	FMI (kg/m^2^)	TFMI (kg/m^2^)	FFMI (kg/m^2^)
SED (min/day)	0.196	0.197	0.203	0.226	0.125
MVPA (min/day)	−0.035	−0.179	−0.164	−0.133	0.007
Total PA (counts/min)	−0.054	−0.199	−0.175	−0.155	−0.006
CRF (mL/min/kg)	−0.380 *	−0.629 *	−0.596 *	−0.558 *	0.119

BMI, body mass index; BF%, body fat percent; FMI, fat mass index; TFMI, trunk fat mass index; FFMI, fat-free mass index; SED, sedentary time; PA, physical activity; MVPA, moderate-to-vigorous PA; CRF, cardiorespiratory fitness. * Statistically significant; *p* < 0.05.

**Table 3:** Associations of physical activity intensities and cardiorespiratory fitness variables in puberty (mean age of 12.1 years) with body composition indices determined by dual-energy X-ray absorptiometry in late adolescence (mean age of 18.1 years).

Pubertal Variable	Late Adolescent Variable				
	BMI (kg/m^2^)	BF%	FMI (kg/m^2^)	TFMI (kg/m^2^)	FFMI (kg/m^2^)
	β *p*	β *p*	β *p*	β *p*	β *p*
SED (min/day) ^a^	−0.117 0.436	0.198 0.347	0.095 0.598	0.139 0.439	−0.325 0.090
MVPA (min/day) ^a^	0.113 0.706	0.780 0.086	0.667 0.113	0.570 0.106	−0.347 0.365
Total PA (conts/min) ^b^	0.098 0.164	−0.051 0.600	−0.008 0.920	−0.038 0.650	0.143 0.122
CRF (mL/min/kg) ^a^	−0.067 0.314	−0.221 0.015 *	−0.212 0.006 *	−0.212 0.015 *	0.102 0.228

The results are presented as a standardized regression coefficient (β), and the *p* value is given for each association. *p <* 0.05 was considered statistically significant. BMI, body mass index; BF%, body fat percent; FMI, fat mass index; TFMI, trunk fat mass index; FFMI, fat-free mass index; SED, sedentary time; PA, physical activity; MVPA, moderate-to-vigorous PA; CRF, cardiorespiratory fitness. ^a^ Adjusted for age, BMI, total PA and total awake accelerometer wear time in puberty. ^b^ Adjusted for age, BMI and total awake accelerometer wear time in puberty. * Statistically significant; *p* < 0.05.

**Table 4:** Mean (±SD) late adolescent values (mean age of 18.1 years) for low and high cardiorespiratory fitness, moderate-to-vigorous physical activity, and body mass index pubertal values (mean age of 12.1 years).

Late Adolescent Variable	Pubertal Variable					
	CRF		MVPA		BMI	
	Low	High	Low	High	Low	High
BMI (kg/m^2^)	23.3 (3.7)	21.4 (1.7) *	22.6 (3.2)	22.2 (2.9)	20.7 (1.8)	23.9 (3.2) *
BF%	28.7 (10.1)	16.5 (6.1) *	19.1 (5.2)	17.3 (4.3)	16.4 (3.5)	19.9 (5.3) *
FMI (kg/m^2^)	4.8 (2.0)	3.3 (0.7) *	4.3 (1.8)	3.8 (1.5)	3.4 (1.0)	4.8 (1.9) *
TFMI (kg/m^2^)	2.1 (1.0)	1.4 (0.3) *	1.9 (0.9)	1.7 (0.7)	1.4 (0.4)	2.1 (0.9) *
FFMI (kg/m^2^)	17.9 (2.1)	17.6 (1.39	17.8 81.8)	17.8 (1.7)	16.9 (1.2)	18.7 (1.7) *
SED (min/day)	636.1 (89.7)	631.2 (83.4)	645.7 (92.2)	622.0 (79.2)	636.8 (76.8)	630.6 (95.2)
MVPA (min/day)	56.7 (28.3)	59.1 (24.9)	51.4 (25.0)	64.2 (26.8) *	57.9 (27.2)	57.8 (26.3)
Total PA (counts/min)	378 (132)	411 (125)	360 (102)	428 (143) *	403 (136)	386 (122)
CRF (mL/min/kg)	42.2 (7.7)	48.5 (7.6) *	44.2 (6.9)	46.3 (9.4)	45.7 (7.8)	44.9 (8.8)

CRF, cardiorespiratory fitness; PA, physical activity; MVPA, moderate-to-vigorous physical activity; BMI, body mass index; BF%, body fat percent; FMI, fat mass index; TFMI, trunk fat mass index; FFMI, fat-free mass index; SED, sedentary time. * Significantly different from Low; *p* < 0.05.

## Data Availability

The data presented in this study are available on a request from the corresponding author for researchers who meet the criteria for access to confidential data.
